# Allelic Variation at Glutenin Loci (*Glu-1*, *Glu-2* and *Glu-3*) in a Worldwide Durum Wheat Collection and Its Effect on Quality Attributes

**DOI:** 10.3390/foods10112845

**Published:** 2021-11-18

**Authors:** Pablo F. Roncallo, Carlos Guzmán, Adelina O. Larsen, Ana L. Achilli, Susanne Dreisigacker, Elena Molfese, Valentina Astiz, Viviana Echenique

**Affiliations:** 1 Centro de Recursos Naturales Renovables de la Zona Semiárida (CERZOS), Departamento de Agronomía, Universidad Nacional del Sur (UNS)-CONICET, Buenos Aires 8000, Argentina; roncallo@cerzos-conicet.gob.ar (P.F.R.); alachilli@cerzos-conicet.gob.ar (A.L.A.); 2 Departamento de Genética, Escuela Técnica Superior de Ingeniería Agronómica y de Montes, Edificio Gregor Mendel, Campus de Rabanales, Universidad de Córdoba, CeiA3, ES-14071 Córdoba, Spain; carlos.guzman@uco.es; 3 Compañía Molinera del Sur, Buenos Aires 8000, Argentina; larsen.adelina@gmail.com; 4 Global Wheat Program, International Maize and Wheat Improvement Center (CIMMYT), El Batán, Texcoco 56237, Edo. Mexico, Mexico; S.Dreisigacker@cgiar.org; 5 CEI Barrow, Laboratorio de Calidad Industrial de Granos (Convenio INTA-Ministerio de Desarrollo Agrario), Buenos Aires 7500, Argentina; molfese.elenarosa@inta.gob.ar; 6 EEA Cesareo Naredo, Instituto Nacional de Tecnología Agropecuaria (INTA), Buenos Aires 6417, Argentina; astiz.valentina@inta.gob.ar

**Keywords:** durum wheat, glutenins, gluten strength, grain protein content, haplotypes, SNPs

## Abstract

Durum wheat grains (*Triticum turgidum* L. ssp. *durum*) are the main source for the production of pasta, bread and a variety of products consumed worldwide. The quality of pasta is mainly defined by the rheological properties of gluten, an elastic network in wheat endosperms formed of gliadins and glutenins. In this study, the allelic variation at five glutenin loci was analysed in 196 durum wheat genotypes. Two loci (*Glu-A1* and *Glu-B1*), encoding for high-molecular-weight glutenin subunits (HMW-GS), and three loci (*Glu-B2*, *Glu-A3* and *Glu-B3*), encoding for low molecular weight glutenin subunits (LMW-GS), were assessed by SDS-PAGE. The SDS-sedimentation test was used and the grain protein content was evaluated. A total of 32 glutenin subunits and 41 glutenin haplotypes were identified. Four novel alleles were detected. Fifteen haplotypes represented 85.7% of glutenin loci variability. Some haplotypes carrying the 7 + 15 and 7 + 22 banding patterns at *Glu-B1* showed a high gluten strength similar to those that carried the 7 + 8 or 6 + 8 alleles. A decreasing trend in grain protein content was observed over the last 85 years. Allelic frequencies at the three main loci (*Glu-B1*, *Glu-A3* and *Glu-B3*) changed over the 1915–2020 period. Gluten strength increased from 1970 to 2020 coinciding with the allelic changes observed. These results offer valuable information for glutenin haplotype-based selection for use in breeding programs.

## 1. Introduction

Durum wheat grains (*Triticum turgidum* L. ssp. *durum* Desf. Husn) are widely consumed all around the world as an important part of the diet in several countries. Historically, it was used worldwide to make pasta and is also the main source for making different products consumed in the Mediterranean basin, in particular flat and leavened bread, couscous and bulgur, as well as freekeh in the WANA (West Asia and North Africa) region [1]. Pasta is also an important part of the food produced and consumed in Latin America. In particular, in Argentina, the consumption of pasta is 8.54 kg per capita per year, the seventh-highest in the world [2], with a durum wheat growing area of 129,255 ha during the crop season 2020/21, which is comparatively smaller than the area occupied by other cereals and oil crops, although it represents the greatest planted area in Latin America. Until the 1970s, Argentina exported high-quality durum wheat mainly to Italy [3]. However, grain quality decreased during the green revolution with the introduction of semi-dwarf wheat from CIMMYT, mainly due to an increase in *Fusarium* susceptibility, which caused a reduction in both the production area and exports.

The level and composition of protein in gluten is the reason why durum wheat is preferred for making pasta [4,5,6], since the gluten quality strongly affects the firmness of pasta after cooking [7,8]. Moreover, consumers prefer pasta products with a strong yellow colour and firmness, resulting in tasty and nutritionally superior food [9]. Gliadins and glutenins are the major storage proteins in wheat endosperm; they form an elastic network called gluten and can be distinguished based on their solubility or insolubility in aqueous alcohols, respectively [10]. The level [11] and composition [12,13] of protein are directly associated with the quality of wheat-derived products. Gliadins are responsible for gluten viscosity and extensibility and are encoded by two loci (*Gli-1* and *Gli-2*) located on the short arms of group 1 (-γ and -ω types) and 6 (-α and -β types) chromosomes [14,15]. Glutenins are associated with the viscoelastic properties of gluten [16]. Two groups of single monomeric glutenins were separated according to their mobility in SDS-PAGE, and were classified as high- (HMW-GS) and low-molecular-weight glutenin subunits (LMW-GS) controlled by orthologous genes located on the long and short arms of the group 1 chromosomes, respectively [17,18]. LMW-GS are encoded by *Glu-2* and *Glu-3* genes [18,19], whereas HMW-GS are encoded by *Glu-1* genes [20]. It was suggested that *Glu-A1* and *Glu-B1* in tetraploid wheat contained sequences encoding x- and y-type subunits as a result of an ancestral duplication [21]. In cultivated durum wheat the *Glu-A1* y subunit is always inactive.

The association between the γ-gliadin 45 and gluten strength was reported in durum wheat [22]. Later, it was demonstrated that linked gene/s coding for low-molecular-weight (LMW) glutenin subunits were mostly responsible for the changes in quality [23]. The LMW subunits are classified according to two models (1 and 2), associated with low and high quality, respectively [24]. The variability and role of high-molecular-weight (HMW) glutenin subunits were examined in wild emmer [25,26], durum [27] and hexaploid wheat subspecies [28,29].

In Argentina, the genetic variability in durum wheat storage proteins was previously evaluated [30,31] but only using a limited number of genotypes. However, a wider screening of modern and historical Argentinian genotypes, as well as worldwide accessions, could help to identify favourable alleles or allele combinations with greater effect on quality. For this purpose, we used a collection composed of South American and Mediterranean germplasms, also including CIMMYT/ICARDA genotypes or derivatives [32]. This collection was previously evaluated for genetic diversity, population structure and linkage disequilibrium patterns, exhibiting high genetic differences between subpopulations, making it possible to trace the origin of the South American germplasm.

The characterisation and availability of glutenin allelic composition could be beneficial to implement its use in marker-assisted selection (MAS) in breeding programs. In the present study, we assessed (i) the allelic variations in five glutenin genes using a worldwide durum wheat collection composed mainly of Argentinian genotypes [32], (ii) the effect of *Glu* haplotypes or individual allelic variants on gluten strength and protein content, (iii) the association of predictive traits and rheological parameters for different glutenin haplotypes using historical datasets, (iv) the changes in gluten composition due to breeding activities between periods or origins. In addition, a detailed description of LMW subunits, not previously available for Argentinian durum wheat, was undertaken.

## 2. Materials and Methods

### 2.1. Plant Material

The total evaluated plant material consisted of 196 durum wheat accessions (*Triticum turgidum* L. ssp. *durum*), mostly representative of the Argentinian breeding programs (85), but also including accessions from Italy (33), Chile (26), France (21), West Africa and North Asia (WANA) (17), CIMMYT (10) and the USA (4) (Supplementary Table S1).

### 2.2. Glutenin Characterisation and Haplotype Analysis

The genetic variability of high- (HMW) and low-molecular-weight (LMW) glutenin loci was evaluated in 196 durum wheat accessions using the sodium dodecyl sulphate (one dimensional SDS-PAGE) methodology, according to the protocol proposed by Peña et al. [33]. For this purpose, 20 mg of whole meal flour was incubated with 0.75 mL of 50% propanol (*v*/*v*) for 30 min in a Thermomixer (Eppendorf, Germany) at 1400 rpm and 65 °C to extract the gliadins, as described in Maryami et al. [34]. Specifically, tubes were centrifuged for 2 min at 10,000 rpm and the supernatant, including the gliadins fraction, was discarded. This step was repeated to remove any remaining gliadins. Subsequently, 100 uL of a solution with DTT at 1.5% (*w*/*v*) with 50 uL of propanol at 50% (*v*/*v*) and 50 uL of Tris-HCl 0.08 M pH 8.0 was added to the pellet. Tubes were mixed with vortex and incubated for 30 min in a Thermomixer at 1400 rpm and 65 °C. After that, the tubes were centrifuged for 2 min at 10,000 rpm with 100 uL of a solution of vinylpyridine at 1.4% (*v*/*v*), prepared with 50 uL of propanol at 50% (*v*/*v*), and 50 uL Tris-HCl 0.08 M pH 8.0, was added. Tubes were mixed again with vortex and incubated in a Thermomixer at 1400 rpm and 65 °C for 15 min. Then, the tubes were centrifuged for 2 min at 13,000 rpm. The supernatant was transferred to a new tube and 180 uL of a solution of Tris-HCl M pH 6.8, 2% SDS (*w*/*v*), 40% glycerol (*w*/*v*), and 0.02% (*w*/*v*) bromophenol blue was added. Tubes were mixed with vortex and incubated for 5 min in a Thermomixer at 1400 rpm at 90 °C, and then were centrifuged for 2 min at 13,000 rpm. From the supernatant, 6 uL was taken and used to run the gels. Separating gels with a concentration of 15% of acrylamide were prepared using 1 M Tris buffer at pH of 8.0 instead of the conventional 8.8. Gels were run at 12.5 mA per gel for 20 h and then stained using Coomasie blue. Five different loci, two encoding HMW glutenins (*Glu-A1*, *Glu-B1*), and three encoding LMW glutenins (*Glu-A3*, *Glu-B3* and *Glu-B2*) were characterised. Allele designations were made following previously proposed rules [35,36]. LMW-GS model classified the allelic combinations as 1 or 2 at *Glu-3* and *Glu-2* loci associated with low and intermediate/high gluten quality, respectively. Glutenin loci haplotypes were constructed considering all possible allele combinations for these five loci and named as Hap_[number].

### 2.3. KASP Markers

Additionally, two KASP markers for the *Gpc-B1* (6BS) locus affecting grain protein content (SNP marker: wMAS000017) and the glutenin locus *Glu-A1* (1AL) (SNP marker: Glu-Ax1/x2*_SNP) were assessed in the entire collection [37]. Allele-specific primers were designed using FAM and VIC tails and are shown in Supplementary Table S2. Three-week-old seedlings obtained from purified seeds were used for DNA extraction following a CTAB protocol [38]. For PCR, a touchdown protocol was used started with a 15 min hot enzyme activation at 94 °C followed by 11 cycles of 94° for 30 s, 65–55 °C for 60 s (−0.8 °C/cycle), 72 °C for 30 s. This was continued with 26 cycles of 94 °C for 30 s, 57 °C for 60 s, 72 °C for 30 s, and a final step at 10 °C. PCR was carried out using 5 μL of volume per well arrayed in a 384 PCR plate. DNA samples were briefly centrifuged and oven-dried at 60 °C for 1 h. SNP-specific KASP reagents, such as the assay mix and the 2X KASP Master mix, including the fluorescent dyes FAM and VIC, were added to dried DNA samples (150 ng/well). PCR-amplified products were subjected to an end-point fluorescence reading using the PHERAstar Plus plate reader from BMG LABTECH. Alleles were assigned based on the differential fluorescence reading using Excel software.

### 2.4. Field Experiments

Seven field trials were conducted during three growing seasons (three in 2011/12, three in 2014/15 and one in 2017/18) at three locations (Cabildo [39°36′ S, 61°64′ W], Barrow [38°20′ S, 60°13′ W] and Pieres [37°46′ S, 58°18′ W]) under rainfed conditions. From the total genotyped accessions (196), 132 and 170 were phenotyped in successive years according to the population size availability in 2011 (132), 2014 (170) and 2017 (170). Plots were arranged using an alpha-lattice design [39] with two repetitions (incomplete blocks, 17 × 8 in 2011 and 10 × 17 in 2014 and 2017). Seed density was adjusted to achieve 300 viable plants per m^2^. Plot size and growing conditions for each experiment are detailed in Supplementary Table S3. Trials were kept free of weeds and diseases and managed according to local practices.

Additional experiments from the National Durum Wheat Experimental Network (NDWEN) or from the Instituto Nacional de Tecnología Agropecuaria (INTA) breeding program, listed in Supplementary Table S4, were used. From these trials, Argentinian genotypes with contrasting glutenin haplotypes were selected. Unbalanced, historical quality datasets were used to assess the glutenin haplotype effect.

### 2.5. Phenotypic Evaluations

Whole-meal flour from each plot was obtained by milling 20 g samples of clean grains in a Cyclotec^®^ 1093 mill (Foss Denmark). The grain protein content (GPC, %, adjusted to 13.5% H°) was determined by near-infrared spectroscopy (NIR Systems DS2500, Foss Denmark). Gluten strength was assessed using the sodium dodecyl sulfate (SDSS) micro-sedimentation test (mm) (adapted from AACC Method 56–70, 1984; for Argentinian strong gluten wheat). The GPC was obtained from each plot in all the trials, while SDSS was only assessed in the 2011 and 2014 field trials.

In the NDWEN or the INTA trials additional quality parameters, which were not evaluated in the collection, were considered. The percentage of gluten, wet and dry gluten, and the gluten index (GI) were obtained according to the IRAM 15864 (Part 2) method and the ICC standard 155 (International Association for Cereal Science and Technology, 1986) method using the Glutomatic system (Perten Instruments, Sweden). Farinograph parameters (Development time and Energy level) were assessed according to Irvine et al. [40] with modifications. A 50 g dough mixer, with constant water hydration (45%) and a fixed mixing time (8 min) were used. Development time (%) = peak height (min)-end height (min)/peak height (min) × 100. Energy level = (peak height (FU)/20) + Area (cm^2^) [41].

### 2.6. Statistical Analyses

Genotype-adjusted means (LSMEANs) were obtained using a mixed linear model (PROC MIXED) with SAS 9.0^®^ software [42], within a maximum restricted probability model (REML). The analysis was performed annually and by location for each trait. For ANOVA and LSMEAN, performed annually, the locations and incomplete blocks within repetitions were considered as random factors and the genotype and repetitions within environment as fixed factors. Analysis of variance (ANOVA) was performed for each trait considering the period, glutenin markers or haplotypes as fixed factors using the INFOSTAT software [43]. The Tukey–Kramer [44] test was implemented to evaluate allele ranking at each locus, while the BSS test [45,46] was applied to analyse the haplotype rank. This test proposed a recursive algorithm based on the combination of a clustering technique and a hierarchical analysis of variance. The combination of the analysis by year and by location was referred to as the environment. For the breeding period effect, the Duncan test (*p* < 0.05) was used.

## 3. Results

One hundred and ninety-six durum wheat genotypes from different origins and periods, including a large set of Argentinian genotypes, three landraces and historical genotypes, were characterised for their glutenin subunit banding patterns by using SDS-PAGE. A total of 32 glutenin subunits from five evaluated loci were identified in the collection (Table 1). The combination of these allelic variants formed a total of 41 glutenin haplotypes (Table 2).

### 3.1. High-Molecular-Weight Glutenin Subunits (HMW-GS)

**Figure 1:**
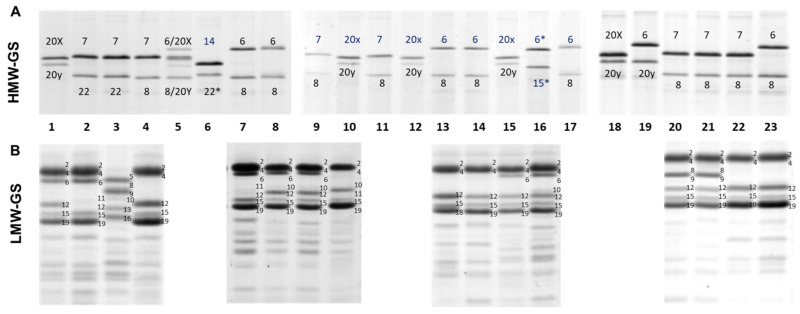
Representative variability of HMW-GS (**A**) and LMW-GS (**B**) found in the evaluated durum wheat collection. Genotypes are as follows: (**A**), 1, Candeal Durumbuck; 2, CRZ-1.12; 3, GAB 125; 4, Gerardo 574; 5, Maristella; 6, Polesine; 7, Buck#33 (33.1123.16-3-4-3); 8, Langdon; 9, Coccorit; 10, Gan; 11, Cham 1 = Waha; 12, Korifla = Cham 3; 13, Focha; 14, Bha; 15, Buck No6; 16, Haurani; 17, Langdon; 18, Cappelli; 19, Capeiti8; 20, Chagual INIA; 21, ACA 5284.06; 22, ACA 3571.13; 23, ACA 3576.13. (**B**), 1, Quc 3462-2009; 2, Quc 3763-2008; 3, Langdon (Dic-3A)-10; 4, CBW 05024; 7, Dupri; 8, Durobonus; 9, Joyau; 10, Biensur; 13, Ardente; 14, Appullo; 15, Ixos; 16, Buck Granate; 20, CBW 0416; 21, CBW 09161; 22, CBW 09270; 23, CBW 09280.

The highest variation between genes encoding for HMW-GS was exhibited by *Glu-B1*, contributing ten alleles to the collection, whereas, for *Glu-A1*, only two alleles were detected (2* (b) and null (c)). The 2* allele at *Glu-A1* was only found in one Argentinian breeding line (CBW 0105), also confirmed by using the KASP marker designed for the *Glu-A1* locus (Supplementary Table S2). At *Glu-B1*, the 7 + 8 (b), 6 + 8 (d) and 20x + 20y (e) alleles covered most of the variation (89.8%) with percentages of 38.3, 26.0 and 25.5, respectively. The frequency of the remaining seven alleles was lower than 4%, as the 7 + 15 (z) subunits detected in seven modern cultivars or breeding lines. The 13 + 16 (f) subunits were only observed in one old Argentinian (Bonaerense 202), one French (Neodur) and three Italians genotypes (Supplementary Table S1). Some specific allelic variants at *Glu-B1* were only detected in Argentinian (subunit 6), Italian (14 + 22* and 6 + 20y) and WANA (6* + 15*) germplasms (Figure 1). The combinations of glutenin subunits 14 + 22*, 6* + 15*, and 6+20y were not previously described and should be considered as novel alleles. We propose to name these alleles *Glu-B1cr*, *Glu-B1cs*, and *Glu-B1ct*, respectively, following the order of the Wheat Gene Catalogue and the latest publications on this topic [34].

### 3.2. Low-Molecular-Weight Glutenin Subunits (LMW-GS)

The highest variation between LMW-GS coding genes was observed at the *Glu-A3* locus with nine alleles, followed by *Glu-B3* and *Glu-B2*, which presented eight and three alleles, respectively (Table 1). The most frequent glutenin subunits encoded by *Glu-A3* were 6 (a) and 6 + 10 (c), with frequencies of 57.7 and 26.5 percent, respectively, followed by null (h) and 5 (b) subunits (Table 1). The *Glu-A3ax* (6.1 subunit) allele was detected privatively in genotypes from Argentina (Buck Cristal and two breeding lines, VF0154 and VF042), but our study conformed a new banding pattern (6.1 + 10). In addition, 10 + 11 and 5 + 10 banding patterns were only detected in the Biensur and Langdon (Dic-3A)-10 cultivars, respectively (Supplementary Table S1). At the *Glu-B3* locus, the *Glu-B3a* (2 + 4 + 15 + 19) allele was present in approximately 90% of the genotypes, followed by 8 + 9 + 13 + 16 and 2 + 4 + 15 + 16 subunit combinations. A new banding pattern (2 + 4 + 8 + 9 + 15 + 19) was detected in three Argentinian breeding lines (Figure 1) and a rare variant (2 + 4 + 16) was obtained in the old cultivar Bonaerense 202 (Supplementary Table S1). The analysis at *Glu-B2* resulted in the detection of three alleles (a, b and c), subunit 12 (a) being present in 93.4% of the genotypes. The 12* allele (c) was the least frequent, only detected in two genotypes from Italy and the WANA region, while the null (b) allele was carried by 11 genotypes and it was more widely distributed among the origins than *Glu-B2c* (Table 1).

### 3.3. Effect of HMW-GS and LMW-GS on Gluten Strength and Protein Content

The relevance of glutenin alleles for improving gluten strength (measured by the SDSS test) and grain protein content (GPC) was assessed in samples from a total of seven field trials (Table 3 and Table 4). ANOVA was conducted, taking into account the characterised alleles, in 132 (2011) and 170 (2014–2017) genotypes phenotypically evaluated for quality traits. Data for the genotypes numbered from 171 to 196 are not available since they were added to the collection after the field trials were conducted. For HMW-GS, only the alleles at the *Glu-B1* locus were associated with highly significant differences (*p* < 0.001) in the SDSS test and significant differences in GPC (in four out of seven trials). This locus explained the 36–41% and 18–21% of the variation in the SDSS test during 2011 and 2014, respectively (Supplementary Table S5). The Glu-A1 locus was practically fixed with only one genotype carrying a differential allele, and it became difficult to test for differences.

Considering LMW-GS, all three loci (*Glu-A3*, *Glu-B3* and *Glu-B2*) showed significant differences between alleles in the SDSS test values in all the evaluated experiments. According to the ANOVA test, the SDSS variance was explained in the following order *Glu-B3* > *Glu-A3* > *Glu-B2* in both years. In addition, the *Glu-A3* locus also significantly affected the grain protein content in BW 2011, PS 2014 and BW 2017, whereas the *Glu-B3* showed a significant effect on the GPC in BW 2011, CA 2014, PS 2014 and BW 2017.

For all loci, the SDSS variance was explained significantly in the following order: *Glu-B1* > *Glu-B3* > *Glu-A3* > *Glu-B2*, in 2011, and *Glu-B3* > *Glu-A3* > *Glu-B1* > *Glu-B2*, in 2014 (Supplementary Table S5). After ANOVA, the rank of alleles was established using the Tukey–Kramer test (*p* < 0.01). Banding patterns 7 + 8, 6 + 8, 7 + 15, 7 + 22 and 6* + 15* at the *Glu-B1* locus were associated with the highest mean values of the SDSS test (Table 3 and Table 4). Additionally, the 13 + 16 allele, carried mainly by European genotypes, reached high SDSS test values in several environments, whereas the 20x + 20y banding pattern showed intermediate values in both evaluated years. In addition, the 14 + 22* banding pattern was associated with a detrimental effect on the SDSS test value, but a high GPC value. On the other hand, the 6, 11, 6 + 11 and 6 + 10 banding patterns at the *Glu-A3* locus were associated with high SDSS test values, their effects being significantly different to other alleles in most of the environments. The worst performance was exhibited by genotypes carrying the subunit 5, followed by the 5 + 10 and 6.1 + 10 banding patterns.

At *Glu-B3*, the 2 + 4 + 15 + 19 (allele a) banding pattern was responsible for significantly increased SDSS test values. On the contrary, genotypes carrying the 8 + 9 + 13 + 16 and 1 + 3 + 14 + 18 banding patterns showed the lowest SDSS test values. A null allele (j) at the *Glu-B3* locus, not previously described in durum wheat, was identified in the Chilean breeding line Quc 3506-2009. This allele was associated with a high SDSS test value. Regarding the effect of the *Glu-B2* alleles on the SDSS test, it was 12 (a) > null (b) > 12* (c), although significant differences were only detected between 12* and null, or 12* and 12 subunits.

### 3.4. HMW-GS and LMW-GS Distribution along Different Periods and Origins

**Figure 2:**
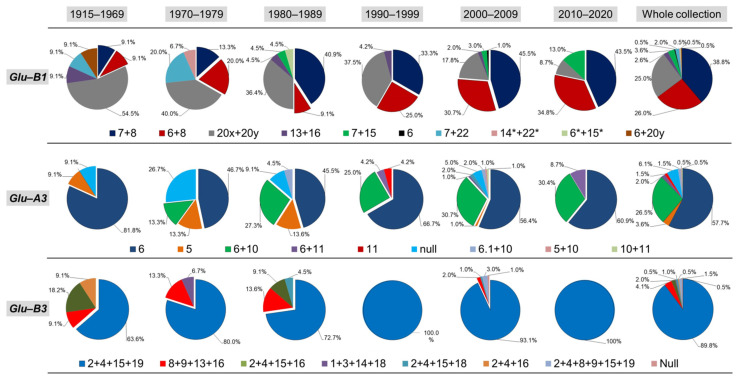
Allelic distribution of the three main glutenin loci (*Glu-B1*, *Glu-A3* and *Glu-B3*) in a worldwide collection of durum wheat over six breeding periods.

Six breeding periods were considered for the analysis of the allele distribution at the three main loci that showed high variability (*Glu-B1*, *Glu-A3* and *Glu-B3*). The results are summarised in Figure 2. The effect of breeding on gluten strength showed a positive selection of alleles associated with high values of the sedimentation test (SDSS). At the *Glu-B1* locus, the allele 20x *+* 20y (e) was associated with low–intermediate gluten quality decreased its frequency over time, whereas the alleles 6 *+* 8 (d) and 7 *+* 8 (b) were favoured by selection and their proportion increased, mostly in the 7 *+* 8 allele. At the *Glu-A3* locus, the proportion of genotypes carrying the allele 6 (a) did not change over time, whereas the genotypes with 6 *+* 10 (c) and 6 *+* 11 (d) increased from the old to the modern germplasm. In addition, the null allele (h) and the subunit 5 (b) were progressively discarded from the 1915–1979 to the 2000–2020 periods. On the other hand, for *Glu-B3* the greatest variability was observed from 1915 to 1979 with a predominance of the 2 *+* 4 *+* 15 *+* 19 (a) allele, which progressively increased to reach 100% in modern genotypes. The allelic distribution by origin is shown in Supplementary Figure S1. At *Glu-B1*, the 6 *+* 8 (d) was the main allele detected in the Argentinian genotypes, while the 7 *+* 8 (b) subunits were more frequent in Chilean and CIMMYT germplasm. The 20x *+* 20y (e) banding pattern was mostly detected in genotypes from France, WANA and Italy. At *Glu-A3*, the subunit 6 was mostly detected in the Argentinian, Italian and Chilean genotypes, whereas 6 *+* 10 (c) was the main allele in germplasm from WANA and France.

### 3.5. Quality Traits Variation over Different Breeding Periods

The variation in GPC and the SDSS test over the six breeding periods previously mentioned was assessed by ANOVA and Duncan tests (*p* < 0.05) for each environment and year. A decreasing trend in GPC was observed from the 1915–1969 to the 2000–2009 periods in six out of seven experiments, with a slight recovery also observed during the 2010–2020 period in some environments (Supplementary Figure S2). This decrease in GPC represented an average of 5.32% (in relative value) from 1934 to 2020 (−0.07% year^−1^ in GPC). On the other hand, the SDSS test values also decreased from 1915–1969 to 1970–1979, but this was later followed by a growth trend in the subsequent periods (Supplementary Figure S2). The overall estimated genetic gain for the gluten strength (SDSS test) was 10.8% from 1934–1969 to 2010–2020 (0.14% year^−1^ in SDSS). However, when the first breeding interval was removed, the overall genetic gain from 1970–1979 to 2010–2020 was 37.8%, representing an increase of 0.48% year^−1^ caused by breeding in our collection.

### 3.6. Glutenin Haplotype Frequency, Distribution and Effect

**Figure 3:**
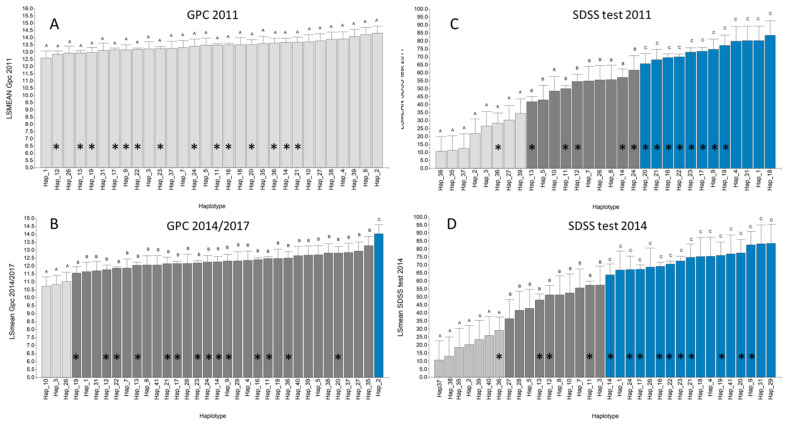
Multiple comparison test (BSS) between haplotypes considering the LSMEANs of grain protein content (GPC) and sodium dodecyl sulfate micro-sedimentation (SDSS) test by year (2011 (**A**,**C**) and 2014 (**D**) or grouping 2014/2017 (**B**)). Bars with a symbol (*) represent haplotypes detected in at least two genotypes. Bars with different letters at the top and colours indicate significant differences according to the BSS test. Hap_: Haplotype_(number).

A total of 41 haplotypes could be conformed based on the alleles detected at the five glutenin loci (Table 2). Most of the haplotypes (30 of total) were poorly represented, showing frequencies of up to 1%. The most frequent haplotype, carried by 22.4% of the genotypes, was Hap_22, followed by Hap_11 and Hap_16 with 12.8% each, Hap 17 (10.2%) and Hap_23 (9.2%). The distribution of haplotypes by origin is shown in Supplementary Table S6. The most frequently observed haplotypes among the genotypes that ranked in the top 20% for the best SDSS test values were Hap_22 (for ex. BonINTA Cumenay), Hap_23 (CBW 09034), Hap_17 (CBW 0111) and Hap_16 (Buck#33). Additionally, some of the less frequent haplotypes obtained high SDSS values in both years (Hap_9, Hap_19, Hap_31, Hap_1, Hap_4 and Hap_18). Significant differences between haplotypes in the SDSS test were observed using the Bautista multiple comparison test *p* < 0.05) (BSS, [45]). A significant haplotype effect on GPC was only observed in three environments (BW 2011, PS 2014 and BW 2017) and the LSMEAN in the 2014/2017 trials (Figure 3). The ANOVA test considering the haplotype effect explained 70–77% of the variance in the SDSS test during 2011, and 48–53% during 2014, whereas, for GPC, it explained 33% of the phenotypic variation (Supplementary Table S5a,b).

### 3.7. Haplotype Effect on Additional Quality Parameters in Argentinian Genotypes

The contrasting haplotype effects in additional quality parameters, such as % gluten, wet gluten, dry gluten, gluten index and farinograph measurements (Development time and Energy level) as well as protein content, were analysed using historical datasets of Argentinian genotypes. For these analyses, 13 genotypes grown in up to 11 environments (11–175) from 1995/96 to 2017/18, representing a total of eight haplotypes (detailed in Supplementary Table S4), were used. These haplotypes varied at the *Glu-B1*, *Glu-B2* and *Glu-A3* loci. The ANOVA test showed a significant effect of the haplotypes on the Gluten Index, Grain protein, Energy level and Development time (Supplementary Figure S3). The haplotypes Hap_23 and Hap_22 exhibited the highest gluten index and Energy level, but obtained the lowest Development time from the farinograph. Additionally, the Hap_29 carrying 20x *+* 20y subunits at *Glu-B1* but a triple null at *Glu-A1*, *Glu-B2* and *Glu-A3* and *Glu-B3a* also showed a high gluten index and Energy level.

## 4. Discussion

The importance of durum wheat prolamins (glutenins and gliadins) and their effect on gluten strength for pasta and breadmaking was mainly studied in different genetic backgrounds [4,7,47,48,49,50,51]. Our study focused on the characterisation of HMW-GS and LMW-GS variation in a worldwide durum wheat collection. The effect of these variants on quality traits was analysed gene-by-gene or as haplotypes, highlighting the importance of common and rare allelic variants to improve gluten quality. A description was also made of the glutenin profile of landraces, as well as old and modern germplasms, mainly from Argentina and other countries.

### 4.1. Glutenin Allelic and Haplotype Variation and Its Effect on GPC and the SDSS Test

In this study, a high number of alleles were detected at the glutenin loci, the *Glu-B1*, *Glu-A3* and *Glu-B3* genes being the main sources of variation in modern durum wheat germplasms exploited for gluten strength improvement. Most of the SDSS variance was explained alternatively by *Glu-B1* or *Glu-B3* in the two years evaluated, indicating that the expression of these genes was affected environmentally. However, these genes exerted a small effect on GPC in one year (2014), suggesting a weak association with this trait. Previous studies yielded controversial results regarding the relative importance of *Glu-B1* and *Glu-B3* and their association with gluten quality. According to Martínez et al. [52], the alleles at *Glu-B3* strongly affect the gluten quality measured by the SDSS, mixograph and alveograph tests. On the contrary, *Glu-B1* was also reported to play a major role in the end-use quality of durum wheat, affecting W and P/L ratio values [53]. However, other authors did not find any effect of *Glu-B1* on gluten quality [54].

A better association between the glutenin loci and quality traits was obtained based on haplotypes, which could explain most of the variation in SDSS, most likely because the haplotype analysis also considers the possible effect of the interactions between loci.

Considering individual loci, *Glu-A1* (HMW-GS) and *Glu-B2* (LMW-GS) showed the lowest variation in our collection. The low variation at *Glu-A1* in modern germplasms was also mentioned by several authors [27,50]. The null allele was practically fixed in our collection as well as in modern germplasms worldwide [51,53,55]. In our analysis, the 2* (b) allele was the unique differential subunit at *Glu-A1* and was also detected at a very low frequency in durum wheat cultivars by other authors [27]. However, frequencies of 18.4% [56], 23.3% [57] and 41.1% [34] were observed in Iranian and Spanish durum wheats and Iranian bread wheat landraces, respectively. We found the 2* subunit to be associated with high gluten strength (higher SDSS), as was previously reported [56,58]. Nonetheless, the contribution of the non-null *Glu-A1* alleles to improve durum wheat quality is still not well defined [55].

In our study, the highest variation was observed at the *Glu-B1* locus with ten alleles, which coincided with previous reports on landraces and modern cultivars of durum wheat [50]. *Glu-B1b* (7 + 8) followed by *Glu-B1d* (6 + 8) and *Glu-B1e* (20x + 20y) were the most frequent alleles. Similar results were obtained in durum wheat, but with different frequencies, with 20x + 20y being > 6 + 8 > 7 + 8 [27]. In this study, the *Glu-B1d* (6 + 8) or *Glu-B1b* (7 + 8) alleles were among the four alleles with higher SDSS values. However, this effect was environmentally impacted, showing *Glu-B1d* with higher SDSS values in 2011 and *Glu-B1b* in 2014. The lower precipitation and mean temperature values obtained at all locations in 2011, suggested that an environmental effect could affect the allelic performance. *Glu-B1b* was widely associated with strong gluten and a good pasta-making quality [53]. Conversely, subunits 6 + 8 were associated with higher SDSS values than 7 + 8 and 20x + 20y [52]. According to Ammar et al. [59] *Glu-B1d* exhibited a better overall breadmaking quality compared to the 7 + 8 or 20 banding patterns. Based on our results, we cannot establish a clear order of importance at *Glu-B1* between the 7 + 8 and 6 + 8 banding patterns or the haplotypes carrying these alleles, but a small difference of *Glu-B1b* (7 + 8) increasing SDSS was observed.

Previously, the 20x + 20y banding pattern was associated with inferior quality [48,59]. In our study, the *Glu-B1e* (20x + 20y) allele was associated with intermediate values of SDSS. This result was coincident with previous studies [31]. However, genotypes with Hap_14 and Hap_29, where *Glu-B1e* was combined with the *Glu-A3h* (null) allele, resulted in similar mean SDSS values to the genotypes with the 6 + 8 banding pattern. The breeding line CBW 05024 (Hap_29) carried *Glu-B1e*/*Glu-A3h*, but also a null allele at *Glu-A1* and *Glu-B2*, which could be an interesting alternative haplotype, showing a high gluten strength.

Two additional *Glu-B1* banding patterns involving the Bx7 subunit (7 + 15 and 7 + 22) were identified among old, intermediate and modern genotypes from Argentina, France, Italy and CIMMYT. *Glu-B1z* (7 + 15) was reported in CIMMYT-derived germplasms and in one Iranian landrace [56,60]. In addition, the *Glu-B1ch* (7 + 22) allele was reported in one Mediterranean landrace [50] and three Iranian landraces [56]. The latter authors also associated *Glu-B1ch* with low gluten quality. Conversely, in the present study the haplotypes (Hap_19, Hap_20 and Hap_21) carrying 7 + 15 or 7 + 22 banding patterns ranked among the best glutenin profiles increasing the SDSS values, suggesting that they should be more carefully evaluated for use in durum wheat breeding programs. The *Glu-B1f* (13 + 16) allele was detected at a low frequency, which was also reported in other cultivars and durum wheat landraces [53,61,62]. However, the frequency of *Glu-B1f* was high (21.38%) in an Algerian durum wheat collection [63] and it was suggested that this allele was associated with low gluten quality [62]. However, our results showed that this allele in Hap_9 was associated with high gluten quality. Hap_9, ranked among the best haplotypes in both years.

A unique banding pattern was carried by the Italian cultivar, Polesine (14 + 22*), associated with low gluten quality (as SDSS), but showing a high GPC value. Two other different banding patterns (13 + 16 and 23 + 18) were previously reported for this genotype [27]. Although the genetics and morphology observed in Polesine, such as plant height and a light green colour (typically only observed in old Italian germplasms) the differences in banding pattern could suggest a misclassification of this material in our seed stock. As far as we know, this is the first report of the 14 + 22* subunit combination in durum wheat. Subunit 22* was previously mentioned as a rare variant [56]. Another unique banding pattern (6* + 15*) was obtained in the landrace, Haurani. A previous study described the 6 + 16 banding pattern in this landrace [60]. Subunit 6* has a higher mobility than subunit 6 in SDS-PAGE. In our study, Haurani showed high SDSS values in both years evaluated, suggesting that the 6* + 15* subunits could be a useful resource for breeding. Additionally, the 6 + 20y banding pattern resulted in a new combination of subunits at the *Glu-B1* locus, only observed in the Italian cultivar, Capeiti8. Nevertheless, different patterns for this cultivar were reported in previous studies as 20x + 20y and 7 + 8 [60,64]. The 6 + 20y banding pattern was not evaluated phenotypically. According to the Catalogue of Gene Symbols for Wheat [65] and the 2020 Supplement (https://wheat.pw.usda.gov/GG3/wgc, accessed date on 2 September 2021), the 14 + 22*, 6 + 20y and 6* + 15* banding patterns were not annotated, and we tentatively propose the nomenclature of *Glu-B1cq*, *Glu-B1cr* and *Glu-B1cs* for these subunit combinations.

Significant interactions between *Glu-B1* and *Glu-B3* or *Glu-A3* were indicated as playing an important effect on quality parameters [52]. Supporting this, the presence of the *Glu-B3b* (8 + 9 + 13 + 16) or *Glu-B3h* (1 + 3 + 14 + 18) allele causes a detrimental effect on SDSS, regardless of which allele is present at *Glu-B1*. Since *Glu-B3a* (2 + 4 + 15 + 19) represented about 90% of the total variation and was gradually fixed in modern germplasms, most of the differences in quality traits were due to the allelic effect from *Glu-B1* and *Glu-A3* and eventually at *Glu-B2*. A low level of variation was observed at *Glu-B3* in comparison with other studies conducted on landraces [50,55,56] or durum wheat germplasm from North Africa [60] and Spain [66]. Contrary to our study, the 2 + 4 + 15 + 18 banding pattern was recorded with a relatively high frequency in Mediterranean landraces [67] and was also reported by other authors [35,56,66].

The rare allelic variant *Glu-B3ag* (2 + 4 + 16) [56] was only detected in the old Argentinian cultivar Bonaerense 202 (1966). In addition, the 2 + 4 + 8 + 9 + 15 + 19 banding pattern was not previously reported, and we tentatively suggest that this new allele be known as *Glu-B3aw*, following the order of the Catalogue of Gene Symbols for wheat [65] and its 2020 Supplement (https://wheat.pw.usda.gov/GG3/wgc, accessed date on 2 September 2021). Additionally, the subunit combination 2 + 4 + 15 + 18 was not previously named and we propose the name of *Glu-B3ax* for this allele.

Another rare variant at *Glu-B3j* (null) was previously mentioned as a result of the wheat–rye translocation 1BL/1RS [68,69] that caused the loss of *Glu-B3* LMW-GS and possibly the linked gliadins (*Gli-B1* locus), and incorporated the secalins (*Sec-1* locus) into the wheat, causing dough stickiness and a reduced gluten quality [70,71,72]. However, the unique genotype which carried *Glu-B3j* (Quc 3506-2009) showed high gluten strength. This Chilean breeding line (Hap_41) is a triple null genotype (*Glu-A1*, *Glu-B3* and *Glu-B2*), also carrying the *Glu-B1b* and *Glu-A3a* alleles, which showed bread wheat ancestry as being the possible origin of the rye translocation. To our knowledge this allele was not previously reported in durum wheat.

Among LMW-GS the *Glu-A3* locus exhibited a slightly higher variability but explained less of the SDSS test variance than *Glu-B3*. The level of polymorphism at *Glu-A3* observed in our study was comparable to previous reports [55,57] in Moroccan and Spanish durum wheat germplasms. The most common variants at *Glu-A3*, 6 and 6 + 10, associated with high gluten strength, are also widely distributed worldwide [36,50,55,56,57,62,66]. However, we could not find a clear significant difference favouring these two alleles over the others. Among the *Glu-A3* alleles, the unique banding patterns 5 + 10 (Langdon (Dic-3A)-10) and 10 + 11 (Biensur) were only previously described in Mediterranean durum wheat germplasms [50]. Only the presence of the subunit 5 (*Glu-A3b*), or combined as 5 + 10, was clearly associated with low SDSS values (Hap_35 to Hap_40). The absence of subunit 5 at *Glu-A3* in old, but also modern, Argentinian genotypes could be one of the reasons for this, since Argentinian durum wheat was considered to be of high quality in the past.

In our study, the 6.1 + 10 banding pattern was only detected in three Argentinian genotypes. The *Glu-A3ax* (6.1) allele was described previously and named in the Argentinian cultivar Buck Cristal [31]. However, we observed the 6.1 + 10 banding pattern in Buck Cristal. This could be attributed to different resolution levels in the methodology. The 6.1 subunit [66] is equivalent to the previously designated 7* subunit [73]. *Glu-A3e* (11) was described in durum wheat by several authors [36,55,62] but only detected in the French and Italian germplasm in our collection.

As previously mentioned, the interaction between HMW-GS and LMW-GS, in particular for the *Glu-1* and *Glu-3* loci, has an important influence on technological properties [74,75,76]. According to He et al., interactions such as *Glu-B1* × *Glu-B3* and *Glu-D1* × *Glu-A3*, strongly affect the SDS sedimentation value, farinograph stability and loaf volume [77]. Similarly, both additive and epistatic effects between glutenin loci significantly affect the dough characteristics [78]. To address this problem, we analysed the effects of these genes on quality traits using haplotypes as in previous reports [33,62,66]. The analysis of allelic combinations instead of individual loci better explained the SDSS and partially explained the GPC variations. Some haplotypes were only detected in the Argentinian (10), Italian (8), French (5), WANA (4), USA (2) and Chilean (1) genotypes, showing the benefits of germplasm exchange. Some rare haplotypes in genotypes from the USA, Italy and Chile were likely a consequence of the allele’s introgression through wide crosses during the breeding process.

### 4.2. Variability in Quality Traits between Breeding Periods and Its Relationship with Allelic Variation at Glutenin Loci

Our results showed a decreasing trend in GPC over six breeding periods in most of the environments evaluated. The negative relationship between GPC and grain yield under most environmental conditions is well known [79,80]. This result agrees well with the increase in grain yield over time, reported by our group [81] using a subset of our collection, as well as from other authors [51,53,82,83]. The estimated reduction in GPC based on our data over time (−0.07% year^−1^) was about half of the value reported in previous studies [53,62,84].

Moreover, the SDSS test showed an initial decrease from the first period (1915–1969) to the second (1970–1979), followed by a consistent improvement in gluten quality until the 2010–2020 period. The positive growth rate period was similar to results obtained for Italian and Spanish cultivars [53,62]. In the same way, an increased glutenin content without any changes in the albumin/globulin rate from 1891 to 2010 was reported in bread wheat [85]. Nevertheless, the improvement in gluten strength over the last 50 years partially recovered the loss observed during the first and second breeding periods. This finding supports the idea that a reduction in grain quality occurred after the introduction of semi-dwarfism during the 1970s. This agrees well with the fact that all genotypes from the 1934–1969 period were tall and carried the Rht-B1a allele [86]. The SDSS genetic gain from 1970–1979 to 2010–2020 (37.8%) was slightly higher than that reported for Spanish and Italian germplasms [62].

Our study shows that the increases in gluten strength over time are strongly associated with changes in allele composition in the HMW and LMW glutenin subunits and were associated with a progressive replacement of the 20x *+* 20y banding pattern by the 7 *+* 8 and 6 *+* 8 subunits at *Glu-B1* as also reported previously [62]. Negative effect alleles, such as *Glu-A3h* and *Glu-A3b*, were also progressively replaced by more advantageous ones (6 *+* 10 and 6 *+* 11). The *Glu-A1*, *Glu-B3* and *Glu-B2* loci were progressively fixed (null(c), 2 *+* 4 *+* 15 *+* 19 (a), 12 (a), respectively) from old to modern accessions, indicating that most of the variation observed today is due to allelic variations at the *Glu-B1* and *Glu-A3* loci. The most frequent haplotypes (22, 23, 17 and 16) carried this conserved allelic combination. The *Glu-B3a*/*Glu-B2a* combination was also present in 75% of all the intermediate and in 100% of the modern Italian cultivars [51].

### 4.3. Haplotype Effect on Additional Quality Parameters

The gluten strength in durum wheat is mainly responsible for pasta quality, and several methodologies were proposed as predictors or as direct tests for rheological properties (micromixograph, viscoelastograph, farinograph, alveograph, gluten index and SDSS) [87]. The SDSS test is widely used as a predictor of gluten strength [24,48,88,89]. Although the SDSS test and Gluten Index are highly correlated, it is noted that the SDSS is clearly influenced by protein content [90]. We evaluated the effect of the five most frequent haplotypes and some contrasting haplotypes in selected Argentinian genotypes by using historical datasets for quality traits, including the gluten index. Our results confirmed that Hap_23 and Hap_22, both carrying 7 + 8 subunits at *Glu-B1*, showed a superior performance based both on the gluten index or farinograph parameters. This analysis provides additional evidence for the high gluten strength associated with Hap_29 by the SDSS test. These results confirm the suitability of SDSS as a gluten strength predictor and its association with allelic variants at glutenin loci and haplotypes.

## 5. Conclusions

In the present study, the allelic variations at five durum wheat glutenin loci were characterised and four new alleles were detected. Additionally, the contribution of individual alleles to improve gluten quality, and their influence on grain protein content, was highlighted, and the haplotype analysis offered valuable information for use in durum wheat breeding programs. Our results showed a decreasing trend in grain protein content over the last 85 years, which could be attributed to a dilution effect due to grain yield improvements. The changes in gluten strength measured by the SDSS test over the same breeding period were associated with the variation in the allele frequency at glutenin loci. Furthermore, some haplotypes poorly represented in modern germplasms were shown to be associated with a high SDSS and quality performance and should be considered for use in breeding programs.

## Figures and Tables

**Table 1:** Allele frequency at five glutenin loci (HMW-GS and LMW-GS) detected in a worldwide collection of 196 durum wheat.

				Argentina (85)		Chile (26)		CIMMYT (10)		France (21)		Italy (33)		USA (4)		WANA (17)	
Locus/Banding Pattern	Allele	N	%	N	%	N	%	N	%	N	%	N	%	N	%	N	%
HMW glutenin subunits																	
*Glu-A1* (2)																	
2 *	b	1	0.5	1	1.2												
null	c	195	99.5	84	98.8	26	100.0	10	100.0	21	100.0	33	100.0	4	100.0	17	100.0
*Glu-B1* (10)																	
7 + 8	b	74	37.8	28	32.9	18	69.2	5	50.0	4	19.0	12	39.4	1	25.0	6	35.3
6 + 8	d	52	26.5	35	41.2	6	23.1	1	10.0	4	19.0	1	3.0	3	75.0	2	11.8
20x + 20y	e	50	25.5	16	18.8	2	7.7	2	20.0	10	47.6	12	33.3			8	47.1
13 + 16	f	5	2.6	1	1.2					1	4.8	3	9.1				
7 + 15	z	7	3.6	3	3.5			2	20.0	2	9.5						
6	an	1	0.5	**1**	**1.2**												
7 + 22	ch	4	2.0	1	1.2							3	9.1				
14 + 22 *	new (cr)	1	0.5									**1**	**3.0**				
6 * + 15 *	new (cs)	1	0.5													**1**	**5.9**
6 + 20y	new (ct)	1	0.5									**1**	**3.0**				
LMW glutenin subunits																	
*Glu-A3* (9)																	
6	a	113	57.7	50	58.8	17	65.4	8	80.0	7	33.3	25	75.8			6	35.3
5	b	7	3.6			1	3.8					2	6.1	1	25.0	3	17.6
6 + 10	c	52	26.5	23	27.1	7	26.9	2	20.0	9	42.9	1	3.0	2	50.0	8	47.1
6 + 11	d	4	2.0	1	1.2	1	3.8			1	4.8	1	3.0				
11	e	3	1.5							2	9.5	1	3.0				
null	h	12	6.1	8	9.4					1	4.8	3	9.1				
6.1 + 10	ax	3	1.5	**3**	**3.5**												
5 + 10		1	0.5											**1**	**25.0**		
10 + 11		1	0.5							**1**	**4.8**						
*Glu-B3* (8)																	
2 + 4 + 15 + 19	a	176	89.8	80	94.1	24	92.3	10	100.0	19	90.5	29	87.9	2	50.0	12	70.6
8 + 9 + 13 + 16	b	8	4.1			1	3.8					2	6.1	2	50.0	3	17.6
2 + 4 + 15 + 16	g	4	2.0	1	1.2							1	3.0			2	11.8
1 + 3 + 14 + 18	h	1	0.5									1	3.0				
2 + 4 + 15 + 18	ax	2	1.0							**2**	**9.5**						
2 + 4 + 16	ag	1	0.5	**1**	**1.2**												
2 + 4 + 8 + 9 + 15 + 19	new (aw)	3	1.5	**3**	**3.5**												
Null	j	1	0.5			**1**	**3.8**										
*Glu-B2* (3)																	
12	a	183	93.4	81	95.3	25	96.2	10	100.0	20	95.2	29	87.9	2	50.0	16	94.1
null	b	11	5.6	4	4.7	1	3.8			1	4.8	3	9.1	2	50.0		
12 *	c	2	1.0									1	3.0			1	5.9
LMW-GS Model																	
1		8	4.1			1	3.8					2	6.1	2	50.0	3	17.6
2		187	95.4	85	100.0	24	92.3	10	100.0	21	100.0	31	93.9	2	50.0	14	82.4
none		1	0.5			1	3.8										
		196	100	85	100.0	26	100.0	10	100.0	21	100.0	33	100.0	4	100.0	17	100.0

Bold number and percentages indicate a unique banding pattern by origin. LMW: Low molecular weight, HMW: High molecular weight. * corresponds with bands showing a slightly mobility difference in SDS-PAGE respect to the original band with the same number.

**Table 2:** Frequency and allele composition of haplotypes for HMW-GS and LMW-GS detected in a worldwide collection of durum wheat.

Loci								
Haplotype	*Glu-A1*	*Glu-B3*	*Glu-B2*	*Glu-B1*	*Glu-A3*	N	Frequency (%)	Quality Traits Assessed ^1^
Hap_1	Ax2 *	2 + 4 + 15 + 19	12	6 + 8	6	1	0.5	yes
Hap_2	null	1 + 3 + 14 + 18	12 *	14 + 22 *	null	1	0.5	yes
Hap_3	null	2 + 4 + 15 + 16	12	20x + 20y	6 + 10	1	0.5	yes
Hap_4	null	2 + 4 + 15 + 16	12	6 * + 15 *	6	1	0.5	yes
Hap_5	null	2 + 4 + 15 + 16	12	20x + 20y	6	1	0.5	yes
Hap_6	null	2 + 4 + 15 + 16	null	6 + 8	6	1	0.5	no
Hap_7	null	2 + 4 + 15 + 18	12	20x + 20y	11	1	0.5	yes
Hap_8	null	2 + 4 + 15 + 18	12	7 + 15	null	1	0.5	yes
Hap_9	null	2 + 4 + 15 + 19	12	13 + 16	6	2	1.0	yes
Hap_10	null	2 + 4 + 15 + 19	12	13 + 16	6 + 10	1	0.5	yes
Hap_11	null	2 + 4 + 15 + 19	12	20x + 20y	6	25	12.8	yes
Hap_12	null	2 + 4 + 15 + 19	12	20x + 20y	6.1 + 10	3	1.5	yes
Hap_13	null	2 + 4 + 15 + 19	12	20x + 20y	6 + 10	11	5.6	yes
Hap_14	null	2 + 4 + 15 + 19	12	20x + 20y	null	3	1.5	yes
Hap_15	null	2 + 4 + 15 + 19	12	6 + 20y	6	1	0.5	no
Hap_16	null	2 + 4 + 15 + 19	12	6 + 8	6	25	12.8	yes
Hap_17	null	2 + 4 + 15 + 19	12	6 + 8	6 + 10	20	10.2	yes
Hap_18	null	2 + 4 + 15 + 19	12	7 + 15	11	1	0.5	yes
Hap_19	null	2 + 4 + 15 + 19	12	7 + 15	6	5	2.6	yes
Hap_20	null	2 + 4 + 15 + 19	12	7 + 22	6	2	1.0	yes
Hap_21	null	2 + 4 + 15 + 19	12	7 + 22	null	2	1.0	yes
Hap_22	null	2 + 4 + 15 + 19	12	7 + 8	6	44	22.4	yes
Hap_23	null	2 + 4 + 15 + 19	12	7 + 8	6 + 10	18	9.2	yes
Hap_24	null	2 + 4 + 15 + 19	12	7 + 8	6 + 11	4	2.0	yes
Hap_25	null	2 + 4 + 15 + 19	12	7 + 8	null	2	1.0	no
Hap_26	null	2 + 4 + 15 + 19	null	13 + 16	11	1	0.5	yes
Hap_27	null	2 + 4 + 15 + 19	null	20x + 20y	6	1	0.5	yes
Hap_28	null	2 + 4 + 15 + 19	null	20x + 20y	6 + 10	1	0.5	yes
Hap_29	null	2 + 4 + 15 + 19	null	20x + 20y	null	1	0.5	yes
Hap_30	null	2 + 4 + 15 + 19	null	6 + 8	6	1	0.5	no
Hap_31	null	2 + 4 + 15 + 19	null	7 + 8	10 + 11	1	0.5	yes
Hap_32	null	2 + 4 + 16	12	13 + 16	6	1	0.5	no
Hap_33	null	2 + 4 + 8 + 9 + 15 + 19	12	6	6	1	0.5	no
Hap_34	null	2 + 4 + 8 + 9 + 15 + 19	12	6 + 8	null	2	1.0	no
Hap_35	null	8 + 9 + 13 + 16	12	20x + 20y	5	1	0.5	yes
Hap_36	null	8 + 9 + 13 + 16	12	7 + 8	5	3	1.5	yes
Hap_37	null	8 + 9 + 13 + 16	12 *	7 + 8	5	1	0.5	yes
Hap_38	null	8 + 9 + 13 + 16	null	20x + 20y	5	1	0.5	yes
Hap_39	null	8 + 9 + 13 + 16	null	6 + 8	5	1	0.5	yes
Hap_40	null	8 + 9 + 13 + 16	null	6 + 8	5 + 10	1	0.5	yes
Hap_41	null	null	null	7 + 8	6	1	0.5	yes
Total (N or %)	2	8	3	10	9	196	100.0	

^1^ Haplotypes reported without quality trait evaluations because they were not among the genotypes assessed in the 2011, 2014 and 2017 field trials. * corresponds with bands showing a slightly mobility difference in SDS-PAGE respect to the original band with the same number.

**Table 3:** The effect of glutenin alleles on grain protein content and the SDSS test in 132 durum wheat genotypes grown in Argentina (2011).

		2011		GPC ^1^				SDSS Test			
Locus/Banding Pattern	Allele	N	%	CA 2011	BW 2011	PS 2011	LSmean	CA 2011	BW 2011	PS 2011	LSmean
HMW glutenin subunits											
*Glu-A1* (2)											
2 *	b	1	0.8	13.92 a	12.29 a	11.5 a	12.58 a	84.50 a	86.66 a	68.51 a	80.17 a
null	c	131	99.2	14.62 a	13.39 a	11.95 a	13.32 a	62.11 a	66.51 a	54.16 a	60.92 a
*Glu-B1* (10)											
7 + 8	b	42	31.8	14.49 a	13.28 a	11.93 a	13.23 a	67.6 b	71.18 b	61.94 b	66.34 b
6 + 8	d	33	25.0	14.81 a	13.32 ab	11.85 a	13.32 a	71.97 b	77.19 b	62.58 b	70.63 b
20x + 20y	e	43	32.6	14.59 a	13.43 ab	11.96 a	13.33 a	48.69 ab	52.66 ab	38.87 ab	46.71 ab
13 + 16	f	4	3.0	14.48 a	13.45 ab	11.78 a	13.23 a	61.0 b	74.37 b	54.24 ab	63.34 b
7 + 15	z	4	3.0	14.57 a	13.49 ab	12.15 a	13.41 a	73.13 b	77.85 b	69.14 b	73.38 b
6	an	na	na	na	na	na	na	na	na	na	na
7 + 22	ch	4	3.0	14.54 a	13.84 ab	12.35 a	13.59 a	69.0 b	71.62 b	60.43 b	66.92 b
14 + 22 *	new (cr)	1	0.8	14.99 a	14.78 b	13.18 a	14.3 a	24.5 a	19.61 a	20.6 a	21.83 a
6 * + 15 *	new (cs)	1	0.8	14.99 a	14.17 ab	12.47 a	13.9 a	80.5 b	88.57 b	70.82 b	79.83 b
6 + 20y	new (ct)	na	na	na	na	na	na	na	na	na	na
LMW glutenin subunits											
*Glu-A3* (9)											
6	a	74	56.1	14.65 a	13.41 ab	11.99 a	13.35 a	64.72 b	68.53 b	56.55 ab	63.28 b
5	b	6	4.5	14.84 a	13.85 ab	12.35 a	13.67 a	22.0 a	22.36 a	18.25 a	20.94 a
6 + 10	c	37	28.0	14.46 a	13.21 ab	11.8 a	13.15 a	64.31 b	70.92 b	56.27 ab	63.83 b
6 + 11	d	1	0.8	15.09 a	13.25 ab	11.94 a	13.4 a	56.5 ab	75.98 b	52.84 ab	61.67 b
11	e	3	2.3	14.6 a	13.23 ab	11.88 a	13.24 a	69.8 b	71.37 b	52.37 ab	64.61 b
null	h	7	5.3	14.94 a	14.14 b	12.4 a	13.84 a	57.0 ab	60.11 ab	48.49 ab	55.0 ab
6.1 + 10	ax	3	2.3	14.09 a	12.64 a	11.0 a	12.57 a	59.2 ab	57.3 ab	54.02 ab	56.83 ab
5 + 10		na	na	na	na	na	na	na	na	na	na
10 + 11		1	0.8	14.34 a	13.01 ab	11.9 a	13.12 a	78.0 b	86.87 b	75.79 b	80.17 b
*Glu-B3* (8)											
2 + 4 + 15 + 19	a	120	90.9	14.59 a	13.34 a	11.9 a	13.28 a	64.9 c	69.6 b	56.79 a	63.78 b
8 + 9 + 13 + 16	b	6	4.5	14.84 a	13.85 ab	12.35 a	13.67 a	22.0 a	22.36 a	18.25 a	20.94 a
2 + 4 + 15 + 16	g	3	2.3	14.41 a	13.69 ab	12.47 a	13.53 a	51.8 abc	52.75 ab	45.42 a	49.78 ab
1 + 3 + 14 + 18	h	1	0.8	14.99 a	14.78 b	13.18 a	14.3 a	24.5 ab	15.5 a	20.6 a	21.83 a
2 + 4 + 15 + 18	ax	2	1.5	15.34 a	13.54 a	12.35 a	13.75 a	58.3 bc	67.07 b	41.03 a	55.25 ab
2 + 4 + 16	ag	na	na	na	na	na	na	na	na	na	na
2 + 4 + 8 + 9 + 15 + 19	new (aw)	na	na	na	na	na	na	na	na	na	na
Null	j	na	na	na	na	na	na	na	na	na	na
*Glu-B2* (3)											
12	a	125	94.7	14.60 a	13.37 a	11.92 a	13.3 a	63.76 b	68.35 b	55.48 b	62.52 b
null	b	5	3.8	14.86 a	13.51 a	12.23 a	13.55 a	42.8 ab	44.22 ab	39.5 ab	42.23 ab
12 *	c	2	1.5	14.47 a	14.08 a	12.84 a	13.78 a	18.75 a	16.93 a	15.59 a	17.17 b
LMW-GS Model											
1		6	4.5	14.6 a	13.85 b	12.35 a	13.67 a	22.0 a	22.4 a	18.3 a	20.9 a
2		126	95.5	14.84 a	13.36 a	11.93 a	13.3 a	64.2 b	68.8 b	56.0 b	60.98 b
none		na	na	na	na	na	na	na	na	na	na

^1^ LSMEAN estimated using 132 genotypes in each environment and over the environments. GPC: grain protein content; SDSS: sodium dodecyl sulfate micro-sedimentation. The environmental references are CA: Cabildo; BW: Barrow; PS: Pieres, followed by the year of sowing. Values in the same column for each locus followed by a different letter are significantly different using the Tukey–Kramer test (*p* < 0.01). * corresponds with bands showing a slightly mobility difference in SDS-PAGE respect to the original band with the same number.

**Table 4:** The effect of glutenin alleles on grain protein content and the SDSS test in 170 genotypes grown in Argentina (2014 and 2017).

		2014/17		GPC ^1^					SDSS Test			
Locus/Banding Pattern	Allele	N	%	CA 2014	BW 2014	PS 2014	BW 2017	LSmean	CA 2014	BW 2014	PS 2014	LSmean
HMW glutenin subunits												
*Glu-A1* (2)												
2 *	b	1	0.6	13.2 a	10.61 a	11.54 a	10.51 a	11.64 a	72.08 a	68.99 a	59.08 a	66.84 a
null	c	161	94.7	12.98 a	12.2 a	12.37 a	11.14 a	12.16 a	58.81 a	66.38 a	66.28 a	63.83 a
*Glu-B1* (10)												
7 + 8	b	65	38.2	12.49 ab	12.31 a	12.31 a	10.93 a	12.0 a	61.99 b	73.03 b	71.16 b	68.69 b
6 + 8	d	44	25.9	13.68 ab	11.94 a	12.34 a	11.23 ab	12.29 a	64.82 b	66.39 ab	67.48 b	66.31 b
20x + 20y	e	47	27.6	13.08 ab	12.26 a	12.41 a	11.3 ab	12.27 a	48.12 ab	55.19 ab	56.46 ab	53.27 ab
13 + 16	f	4	2.4	11.73 ab	11.6 a	11.98 a	11.23 ab	11.59 a	59.34 ab	77.87 b	78.02 b	71.61 b
7 + 15	z	4	2.4	12.32 ab	11.81 a	12.42 a	10.94 a	11.9 a	61.59 b	69.14 b	78.82 b	69.63 b
6	an	na	na									
7 + 22	ch	4	2.4	14.04 ab	12.58 a	12.33 a	11.26 ab	12.48 ab	74.9 b	80.61 b	72.01 b	76.15 b
14 + 22 *	new (cr)	1	0.6	15.38 b	12.5 a	14.82 b	12.7 b	14.02 b	18.41 a	21.97 a	21.28 a	20.31 a
6 * + 15 *	new (cs)	1	0.6	11.43 a	13.31 a	13.67 b	11.82 ab	12.35 a	68.67 b	87.02 b	72.0 b	75.48 b
6 + 20y	new (ct)	na	na									
LMW glutenin subunits												
*Glu-A3* (9)												
6	a	98	57.6	12.98 a	12.23 a	12.39 ab	11.11 ab	12.07 a	61.92 abc	69.18 bc	70.11 b	67.10 c
5	b	6	3.5	13.06 a	13.12 a	13.05 ab	11.69 ab	12.77 a	18.72 a	19.9 a	23.6 a	20.66 a
6 + 10	c	48	28.2	12.89 a	12.08 a	12.23 ab	11.08 ab	12.07 a	58.11 abc	68.68 bc	64.69 ab	63.81 bc
6 + 11	d	2	1.2	13.80 a	11.97 a	12.44 ab	10.73 ab	12.24 a	67.58 bc	73.09 c	60.76 ab	67.15 c
11	e	3	1.8	11.92 a	11.77 a	12.03 ab	11.42 ab	11.78 a	61.25 abc	67.0 abc	72.51 b	66.55 c
null	h	8	4.7	13.35 a	12.15 a	12.65 ab	11.44 ab	12.43 a	57.36 abc	64.48 abc	64.27 ab	62.03 bc
6.1 + 10	ax	3	1.8	13.86 a	11.29 a	11.15 a	10.8 ab	11.72 a	51.01 abc	42.33 abc	58.57 ab	50.7 abc
5 + 10		1	0.6	14.24 a	11.99 a	11.96 ab	12.3 ab	12.64 a	31.45 ab	21.96 ab	23.78 a	25.97 ab
10 + 11		1	0.6	11.79 a	12.33 a	13.29 b	10.42 a	11.69 a	78.4 c	80.56 c	90.74 b	83.22 c
*Glu-B3* (8)												
2 + 4 + 15 + 19	a	156	117.3	12.99 a	12.15 a	12.32 a	11.10 ab	12.13 a	61.17 ab	68.92 bc	68.42 b	66.18 b
8 + 9 + 13 + 16	b	7	5.3	13.23 a	12.96 a	12.98 ab	11.78 ab	12.75 ab	20.54 a	20.20 a	23.63 a	21.42 a
2 + 4 + 15 + 16	g	3	2.3	11.96 a	12.32 a	12.26 a	11.32 ab	11.95 a	49 ab	67.52 abc	58.82 ab	58.59 ab
1 + 3 + 14 + 18	h	1	0.8	15.38 b	12.5 a	14.82 b	12.70 b	14.02 b	18.41 a	21.97 ab	21.88 a	20.31 a
2 + 4 + 15 + 18	ax	2	1.5	10.92 a	12.54 a	12.81 ab	10.88 ab	11.96 a	45.83 ab	44.0 abc	70.91 b	53.48 ab
2 + 4 + 16	ag	na	na									
2 + 4 + 8 + 9 + 15 + 19	new (aw)	na	na									
Null	j	1	0.8	14.61 ab	10.97 a	12.11 a	10.60 a	12.06 a	66.5 b	82.04 c	81.69 b	76.94 b
*Glu-B2* (3)												
12	a	159	93.5	12.96 a	12.19 a	12.33 a	11.11 a	12.14 a	60.06 b	68.02 b	67.54 b	65.22 b
null	b	9	5.3	13.16 a	11.96 a	12.44 a	11.36 a	12.25 a	48.00 b	48.89 b	54.30 b	50.31 b
12 *	c	2	1.2	14.08 a	12.88 a	14.48 b	12.07 a	13.43 b	14.65 a	16.43 a	16.66 a	15.52 a
LMW-GS Model												
1		7	4.1	13.23 a	12.96 b	12.98 a	11.78 a	12.74 a	20.54 a	20.20 a	23.63 a	21.42 a
2		162	95.3	12.96 a	12.16 ab	12.34 a	11.11 a	12.14 a	60.5 b	68.3 b	67.98 b	65.6 b
none		1	0.588	14.61 a	10.88 a	12.11 a	10.60 a	12.06 a	66.5 b	82.04 b	81.68 b	76.94 b

^1^ LSMEAN estimated using 132 genotypes in each environment and over the environments. GPC: grain protein content; SDSS: sodium dodecyl sulfate micro-sedimentation. The environmental references are CA: Cabildo; BW: Barrow; PS: Pieres, followed by the year of sowing. Values in the same column for each locus followed by a different letter are significantly different using the Tukey–Kramer test (*p* < 0.01). * corresponds with bands showing a slightly mobility difference in SDS-PAGE respect to the original band with the same number.

## Data Availability

Data in this study are available in the article and Supplementary Materials. Plant material and raw data are available upon request from the first author.

## Supplementary Materials

The following are available online at https://www.mdpi.com/article/10.3390/foods10112845/s1, Figure S1: Allelic distribution of the three main glutenin loci (*Glu-B1*, *Glu-A3* and *Glu-B3*) considering the origin of accession in the whole collection. Figure S2: Multiple comparison tests (Duncan test, *p* < 0.05) performed on the GPC and SDSS means considering six breeding periods in each environment and by year. The environmental references are CA: Cabildo; BW: Barrow; PS: Pieres, followed by the year of sowing. Bars in the same environment with a different letter on the top are significantly different. Figure S3: Bar plot showing additional quality traits mean values according to the corresponding haplotype. Means with a different letter on the top indicate significant differences according to the Tukey–Kramer test (*p* < 0.05). Table S1: Allelic characterisation of HMW-GS and LMW-GS glutenin loci in a worldwide durum wheat collection of 196 genotypes. Table S2: List of KASP markers for *Glu-A1* and *Gpc-B1* used in this study. Table S3: Precipitation, mean temperature and adopted management in the seven field trials. Table S4: Summary of genotypes/haplotypes and trials considered with additional quality parameters. Table S5: Percentage of variance explained by each locus, model and haplotypes for GPC and SDS sedimentation test. Table S6: Frequency and distribution of haplotypes according to the origin of accessions.
