# Supplementary material for: Allelic Variation at Glutenin Loci (Glu-1, Glu-2 and Glu-3) in a Worldwide Durum Wheat Collection and Its Effect on Quality Attributes
Source: Foods. 2021 Nov 18;10(11):2845. doi: 10.3390/foods10112845 (PMC8623136; doi:10.3390/foods10112845)
Supplement: Supplementary file 1 [file foods-10-02845-s001.zip › Supplementary Figure S1.pptx]

## Slide 1
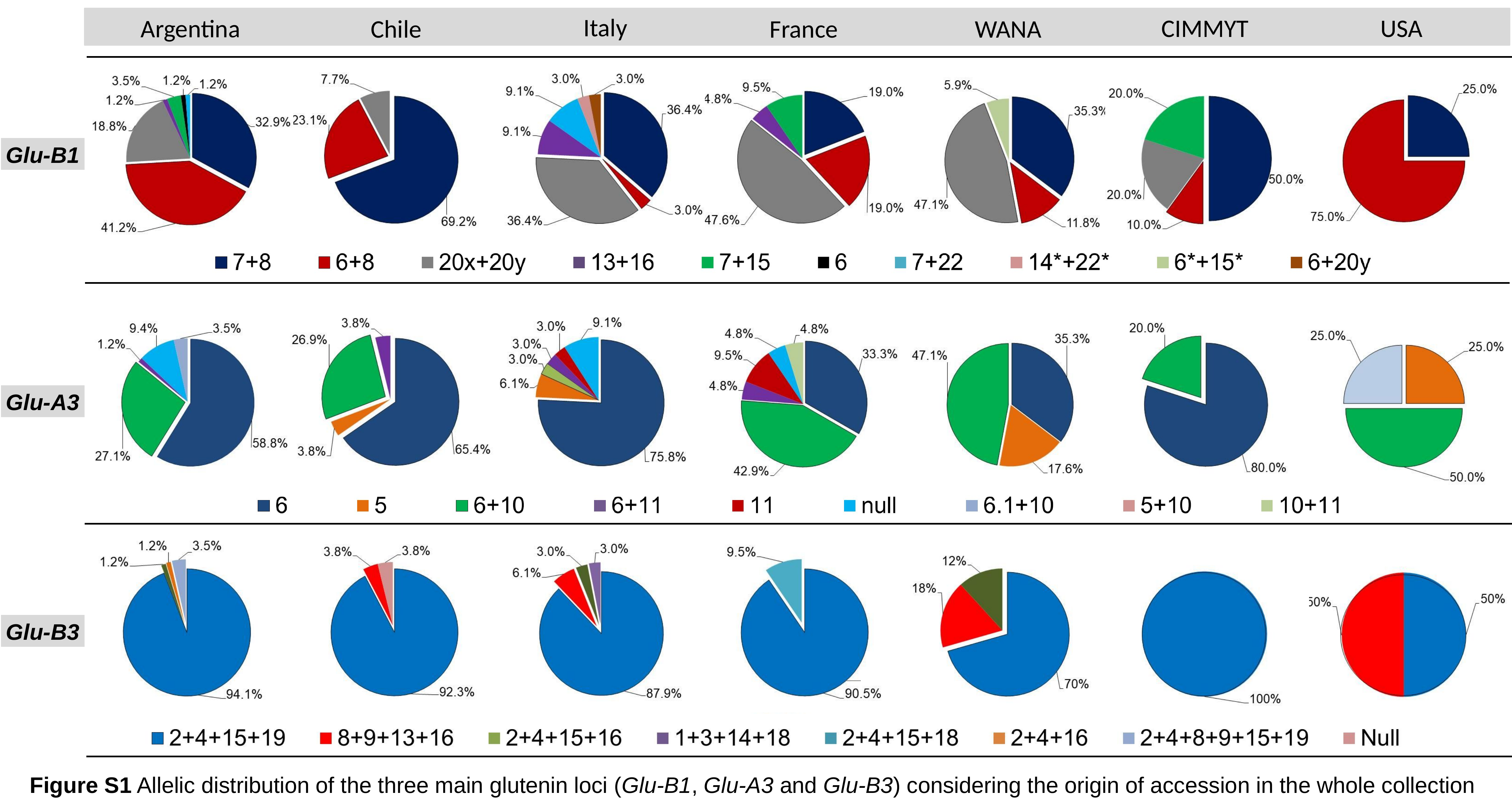

Italy
CIMMYT
USA
 Argentina
WANA
Chile
France
Glu-B1
Glu-A3
Glu-B3
Figure S1 Allelic distribution of the three main glutenin loci (Glu-B1, Glu-A3 and Glu-B3) considering the origin of accession in the whole collection
