# Supplementary material for: Allelic Variation at Glutenin Loci (Glu-1, Glu-2 and Glu-3) in a Worldwide Durum Wheat Collection and Its Effect on Quality Attributes
Source: Foods. 2021 Nov 18;10(11):2845. doi: 10.3390/foods10112845 (PMC8623136; doi:10.3390/foods10112845)
Supplement: Supplementary file 1 [file foods-10-02845-s001.zip › Supplementary Figure S2.pptx]

## Slide 1
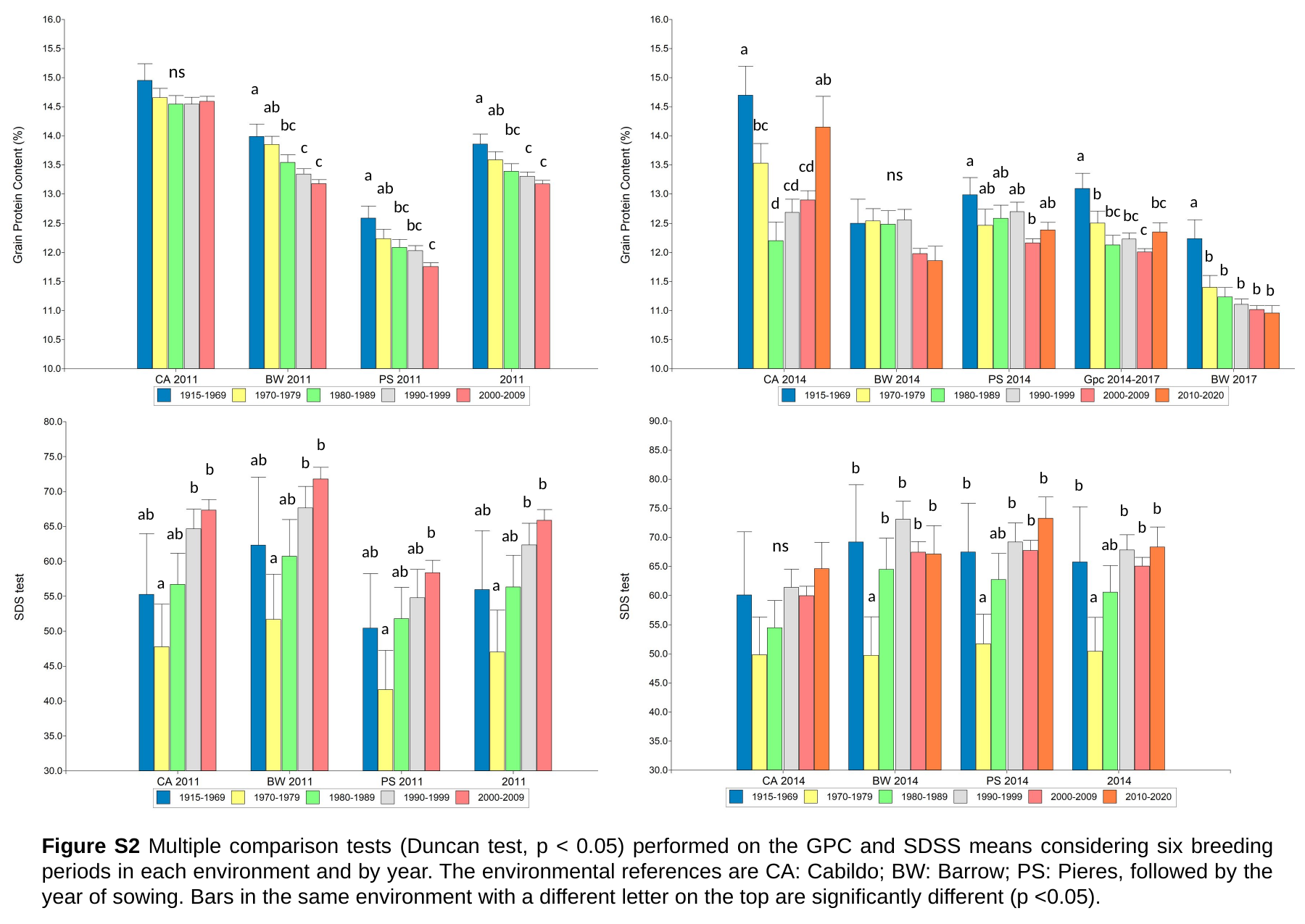

a
ns
ab
a
a
ab
ab
bc
bc
bc
c
c
a
a
c
c
cd
ab
ns
a
cd
ab
ab
ab
b
a
ab
bc
d
bc
bc
bc
b
bc
c
c
b
b
b
b
b
b
ab
b
b
b
b
b
b
b
b
b
ab
b
b
b
b
ab
b
ab
b
b
b
b
b
ab
ab
ab
ab
ns
ab
ab
a
ab
a
a
a
a
a
a
Figure S2 Multiple comparison tests (Duncan test, p < 0.05) performed on the GPC and SDSS means considering six breeding periods in each environment and by year. The environmental references are CA: Cabildo; BW: Barrow; PS: Pieres, followed by the year of sowing. Bars in the same environment with a different letter on the top are significantly different (p <0.05).
