# Supplementary material for: Allelic Variation at Glutenin Loci (Glu-1, Glu-2 and Glu-3) in a Worldwide Durum Wheat Collection and Its Effect on Quality Attributes
Source: Foods. 2021 Nov 18;10(11):2845. doi: 10.3390/foods10112845 (PMC8623136; doi:10.3390/foods10112845)
Supplement: Supplementary file 1 [file foods-10-02845-s001.zip › Supplementary Figure S3.pptx]

## Slide 1
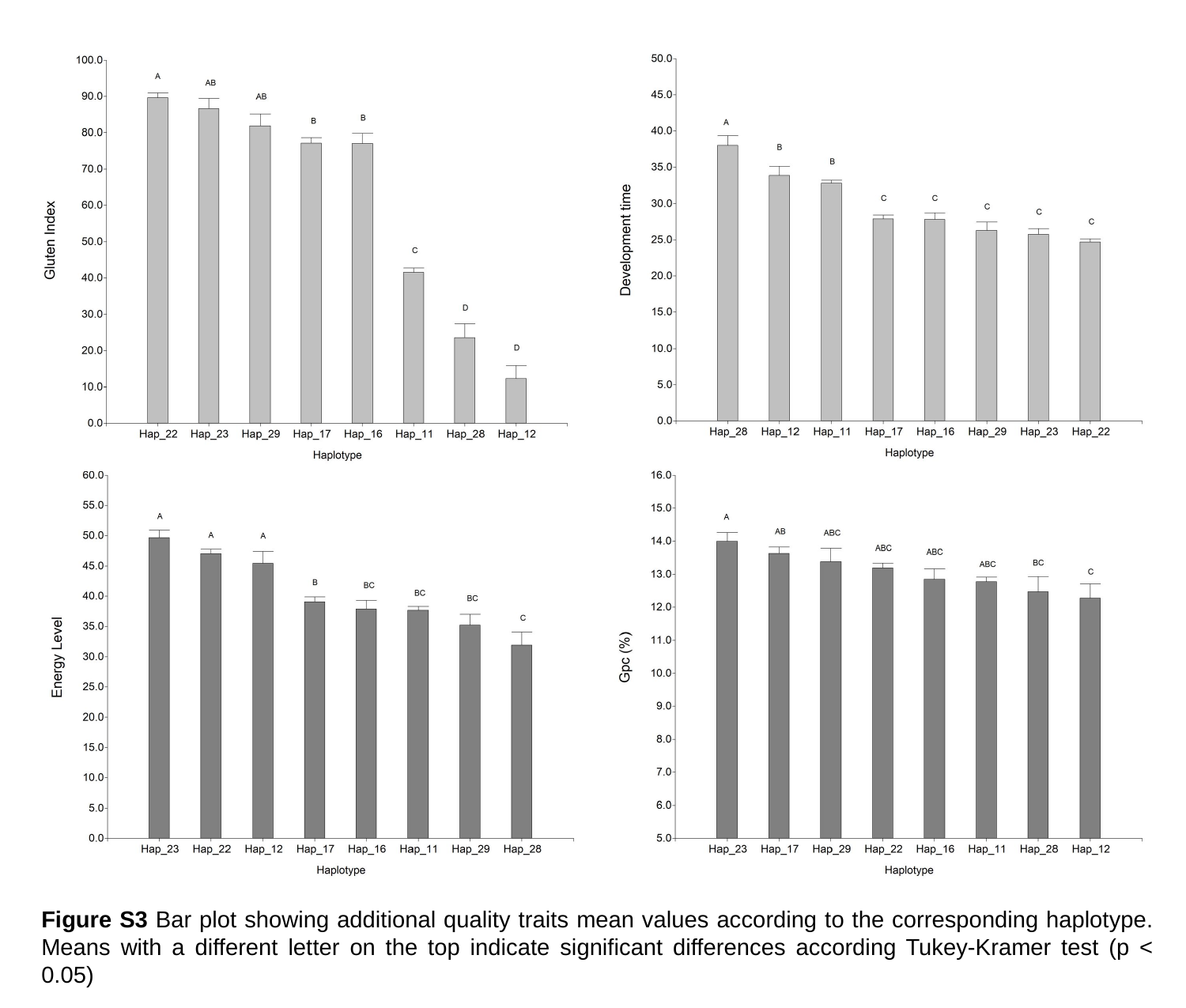

Figure S3 Bar plot showing additional quality traits mean values according to the corresponding haplotype. Means with a different letter on the top indicate significant differences according Tukey-Kramer test (p < 0.05)
